# Modification of a Putative Third Sodium Site in the Glycine Transporter GlyT2 Influences the Chloride Dependence of Substrate Transport

**DOI:** 10.3389/fnmol.2018.00347

**Published:** 2018-09-24

**Authors:** Cristina Benito-Muñoz, Almudena Perona, David Abia, Helena G. dos Santos, Enrique Núñez, Carmen Aragón, Beatriz López-Corcuera

**Affiliations:** ^1^Departamento de Biología Molecular, Universidad Autónoma de Madrid, Madrid, Spain; ^2^Centro de Biología Molecular, “Severo Ochoa" Consejo Superior de Investigaciones Científicas-Universidad Autónoma de Madrid, Madrid, Spain; ^3^Smartligs, Parque Científico de Madrid, Campus de Cantoblanco, Madrid, Spain; ^4^Centro de Investigación Biomédica en Red de Enfermedades Raras, Instituto de Salud Carlos III, Madrid, Spain; ^5^IdiPAZ-Hospital Universitario La Paz, Universidad Autónoma de Madrid, Madrid, Spain

**Keywords:** GlyT, hyperekplexia, sodium site, neurotransmitter reuptake, SLC6

## Abstract

Neurotransmitter removal from glycine-mediated synapses relies on two sodium-driven high-affinity plasma membrane GlyTs that control neurotransmitter availability. Mostly glial GlyT1 is the main regulator of glycine synaptic levels, whereas neuronal GlyT2 promotes the recycling of synaptic glycine and supplies neurotransmitter for presynaptic vesicle refilling. The GlyTs differ in sodium:glycine symport stoichiometry, showing GlyT1 a 2:1 and GlyT2 a 3:1 sodium:glycine coupling. Sodium binds to the GlyTs at two conserved Na^+^ sites: Na1 and Na2. The location of GlyT2 Na3 site remains unknown, although Glu650 has been involved in the coordination. Here, we have used comparative MD simulations of a GlyT2 model constructed by homology to the crystalized DAT from *Drosophila melanogaster* by placing the Na3 ion at two different locations. By combination of *in silico* and experimental data obtained by biochemical and electrophysiological analysis of GlyTs mutants, we provide evidences suggesting the GlyT2 third sodium ion is held by Glu-250 and Glu-650, within a region with robust allosteric properties involved in cation-specific sensitivity. Substitution of Glu650 in GlyT2 by the corresponding methionine in GlyT1 reduced the charge-to-flux ratio to the level of GlyT1 without producing transport uncoupling. Chloride dependence of glycine transport was almost abolished in this GlyT2 mutant but simultaneous substitution of Glu250 and Glu650 by neutral amino acids rescued chloride sensitivity, suggesting that protonation/deprotonation of Glu250 substitutes chloride function. The differential behavior of equivalent GlyT1 mutations sustains a GlyT2-specific allosteric coupling between the putative Na3 site and the chloride site.

## Introduction

Glycine fulfills the fast inhibitory transmission in caudal areas of the adult central nervous system, and it is involved in the processing of motor, sensory, and nociceptive information (Legendre, 2001). In addition, glycine exerts positive modulation of excitatory transmission acting as a co-agonist of glutamate on *N*-methyl-D-aspartate receptors (Johnson and Ascher, 1987). Clearance of synaptic glycine is accomplished by two specific plasma membrane GlyTs – GlyT1 and GlyT2 (Aragon and Lopez-Corcuera, 2003). GlyT1, mainly present in astrocytes, is the main regulator of glycine synaptic levels (Gomeza et al., 2003a). The neuronal GlyT2 supplies neurotransmitter for presynaptic vesicle refilling and promotes the recycling of synaptic glycine at inhibitory synapses (Rousseau et al., 2008). The absence of GlyT2 function eliminates inhibitory glycinergic neurotransmission (Gomeza et al., 2003b). In humans, this condition causes hyperekplexia or startle disease, a rare disorder characterized by exaggerated startle responses to trivial stimuli that may have severe consequences due to apnea episodes (Rees et al., 2006; Gimenez et al., 2012; Arribas-González et al., 2015). Moreover, GlyTs are modulated by pain mediators and are potential pharmacological targets for pain therapy (Zeilhofer et al., 2005; Harvey and Yee, 2013).

GlyT1 and GlyT2 are members of the human SLC6 family of sodium- and chloride-dependent neurotransmitter transporters (NSS) together with the GABA, and the monoamine transporters (Rudnick et al., 2014). GlyTs protein fold was first described in the leucine transporter from *Aquifex aeolicus* (LeuT_Aa_, PDB code 2A65) (Yamashita et al., 2005) and contains two topologically inverted structural repeats of five TMs. The repeats intertwine to form two bundles that rock one against the other during transport: a scaffold bundle (TMs 3,4,8,9) and a mobile core bundle (TMs 1,2,6,7). Thus, the central substrates-binding pocket gains alternatively access to either side of the membrane via conformational changes (Jardetzky, 1966; Shi, 2013; Rudnick et al., 2014). LeuT_Aa_ has been presently crystalized in outwardly facing, occluded, inwardly facing conformations, and in the presence of several inhibitors (Yamashita et al., 2005; Zhou et al., 2007, 2009; Singh et al., 2008; Krishnamurthy and Gouaux, 2012). Later, the eukaryotic transporters for dopamine from *Drosophila melanogaster* (*d*DAT) (Penmatsa et al., 2013, 2015) and for serotonin from human (*h*SERT) (Coleman et al., 2016) were crystalized in inhibitor-bound forms.

Molecular dynamics simulations of GlyT structures modeled using LeuT_Aa_ crystalographic structure as a template predicted the conservation of the substrate (glycine) and two sodium-binding sites Na1 and Na2 (Perez-Siles et al., 2012). The use of these sites by the GlyTs was confirmed through site-directed mutagenesis and analysis of GlyTs sensitivity to lithium ion (Perez-Siles et al., 2011, 2012). GlyT1 and GlyT2 differ in their sodium:glycine symport stoichiometry which is 2:1 for GlyT1 and 3:1 for GlyT2 (Roux and Supplisson, 2000), and this property is crucial for their unique physiological roles (Gomeza et al., 2003a,b). A transitory cation binding site was identified in the extracellular vestibule of GlyT2 (Perez-Siles et al., 2012) that partially overlaps with a transient Na^+^-binding site lately discovered in LeuT_Aa_ (Zomot et al., 2015). However, although a proposal was recently made, the location of the GlyT2 Na3 site remains elusive (Subramanian et al., 2016). The chloride binding site was predicted to be adjacent to the Na1 site in the eukaryotic chloride-dependent transporters (Zomot et al., 2007; Kantcheva et al., 2013; Penmatsa et al., 2013). Still, the relationship between sodium and chloride sites and the role of chloride in GlyT2 transport cycle is unsettled. The structural dynamics and interactions of GlyT2 may provide valuable information for the study of pathological aspects of GlyTs such as hyperekplexia.

## Materials and Methods

### GlyTs Mutagenesis and Transporter Expression

Substitution mutants were generated with the QuikChange II Site-Directed Mutagenesis kit (Stratagene, La Jolla, CA, United States), using the rGlyT2 (Liu et al., 1993) subcloned in pCDNA3 as reported (Arribas-Gonzalez et al., 2013) or rGlyT1 (Smith et al., 1992) in pcDNA3 (Perez-Siles et al., 2012). The complete coding region of the constructs was sequenced to verify that only the desired mutation had been introduced. Plasmids from two independent *Escherichia coli* colonies were expressed into eukaryotic cells and [^3^H]glycine transport was measured for verification. COS7 cells (American Type Culture Collection, Rockville, MD, United States) were grown and transfected using TurboFect Transfection Reagent (Thermo Fisher Scientifics, Waltham, MA, United States), following the manufacturer’s protocol (2 μl reagent/μg of DNA). Cells were incubated for 48 h at 37°C until used (Arribas-Gonzalez et al., 2015).

### Transport Assays

COS7 cells were washed and incubated at 37°C in HBS (in mM: 150 NaCl, 10 HEPES-Tris, pH 7.4, 1 CaCl_2_, 5 KCl, 1 MgSO_4_, 10 glucose) containing 2 μCi/ml [2-^3^H]glycine (1.6 TBq/mmol; PerkinElmer Life Sciences, Boston, MA, United States), at 10 μM final glycine concentration if not otherwise stated (Gimenez et al., 2012). At the end of the desired time (10 min), reactions were washed and terminated by aspiration. Protein concentration (Bradford, Biorad, Berkeley, CA, United States) and [2-^3^H]glycine (liquid scintillation, Opti-Fluor, PerkinElmer Life Sciences, Boston, MA, USA, LKB 1219 Rackbeta) were determined. Glycine accumulation by mock-transfected cells was subtracted from that of the transporter-transfected cells and normalized by the protein concentration. Kinetic analyses were performed by varying glycine concentration in the uptake medium between 0.5 and 500 μM.

### Surface Biotinylation

Cells expressing the transporters were labeled with 1.0 mg/ml Sulfo-NHS-Biotin (Pierce, Cambrige, MA, United States) in PBS at 4°C, as described (Arribas-Gonzalez et al., 2015). Free biotin quenching was performed by adding 100 mM L-lysine in PBS and then 1× lysis buffer: in mM, 150 NaCl, 50 Tris-HCl pH 7.4, 5 EDTA, 0.4 PMSF supplemented with 1% Triton X-100, 0.1% SDS, 0.25% sodium deoxycholate, and 4 μl/ml protease inhibitor cocktail (Sigma-Aldrich, St. Louis, MO, United States). A lysate aliquot was saved (total protein content), and the remainder was incubated with 50% streptavidin–agarose beads (Sigma-Aldrich, St. Louis, MO, United States) for 2 h at room temperature and centrifuged. An aliquot of the supernatant was saved to quantify the non-biotinylated fraction and the agarose beads were recovered, washed three times with 1× lysis buffer, and bound proteins (biotinylated) were eluted with Laemmli buffer (65 mM Tris, 10% glycerol, 2.3% SDS, 100 mM DTT, 0.01% bromophenol blue) for 10 min at 75°C. Samples were analyzed by Western blot as described (Arribas-Gonzalez et al., 2015).

### Homology Modeling of GlyT2

The crystalized DAT from *d*DAT (PDB code 4M48) (Penmatsa et al., 2013) was used as the homology model template. Alignments between the sequence of *d*DAT and human GlyT2 (Q9Y345.3| SLC6A5_HUMAN) were obtained by profile-profile alignment using the program Muscle (Edgar, 2004). Profiles for the protein sequence were obtained by MSA using the program MAFFT (Katoh et al., 2002). Sequences included in MSAs were detected by the program blastp (Altschul et al., 1990) against the NCBI’s nr database, with an e-value below 1e-15. A GlyT2 model with the bound substrate (glycine) and coupled ions was obtained using MODELER version 9.13 (Sali and Blundell, 1993). Initial alignments were iteratively refined based on the evaluation of obtained model. The modeled region for GlyT2 was from Lys191 to Trp760, excluding the loop between Leu329 and Ala365, which is not present in *d*DAT. Mutants were built using the Mutagenesis Wizard implemented in PyMol (The PyMOL Molecular Graphics System). Systems setup entailed adding hydrogen atoms, to assign atom types and charges according to AMBER ff03 force field (Duan et al., 2003), and to determine the protonation state of ionizable residues at pH 7 using H++ web server^1^ (Gordon et al., 2005).

### Molecular Dynamics Simulations

The three 3D models (GlyT2-Na3 location 1 model, GlyT2-Na3 location 2 model, and GlyT2-E650M model) were further refined by means of MD simulations. Using the Membrane Builder module (Jo et al., 2007, 2009) in CHARMM-GUI ^2^ (Jo et al., 2008), each protein was inserted in a pre-equilibrated box containing a POPC lipid bilayer, POPE lipid bilayer, cholesterol molecules (in a proportion 2:2:1) covered by water up and down, and a 0.15-M concentration of Na^+^ and Cl^-^ ions. We performed MD simulations using AMBER12 (Case et al., 2012). The protocol was as follows: (i) energy minimization to relax the initial model (20 ps); (ii) heating (100 k), holding lipid molecules, and Cα carbons fixed (100 ps); (iii) heating (300 k), holding lipid molecules, and Cα carbons fixed (100 ps); (iv) holding, to equilibrate the water molecules around the solutes (10 × 200 ps, lowering restraints on lipid molecules and Cα carbons); (v) after successfully equilibration, production at constant temperature and pressure (50 ns). All the simulations were performed at constant pressure (1 atm) and temperature (300°K) with an integration time step of 2 fs. The SHAKE algorithm (Ryckaert et al., 1977) was used to constrain all the bonds involving H atoms at their equilibrium distances. Periodic boundary conditions and the particle mesh Ewald methods were applied to treat long-range electrostatic effects (Darden et al., 1993). AMBER ff03 (Duan et al., 2003), Lipid11 (Skjevik et al., 2012), Lipid14 (Dickson et al., 2014), and TIP3P (Jorgensen et al., 1983) force fields were used in all cases.

### Analysis of MD Trajectories

The stability of each complex was evaluated by calculating the root-mean-square-deviation (RMSD) of the Cα atoms along the trajectories, using their starting structures as reference. Additionally, the root-mean-square-fluctuation (RMSF) of each residue, relative to the corresponding average value, was calculated once each snapshot had been fitted to its initial structure. The effective binding free energies between the ligands and the more relevant residues in the binding site were qualitatively estimated using the MM/GBSA (Miller et al., 2012; Genheden and Ryde, 2015). MM/GBSA is a popular approach to estimate the free energy of the binding of small ligands to biological macromolecules. MM/GBSA takes into account a MM interaction term, a solvation contribution thorough a generalized born (GB) model, and a surface area (SA) contribution to account for the non-polar part of desolvation. The MM part estimates the enthalpic contributions for the protein–ligand interactions (bonded, electrostatic, vanderWaals). The polar solvation energy represents the electrostatic interaction between the solute and the continuum solvent. In addition, three non-polar solvation terms include cavitation, dispersion, and repulsion energies, representing the cost of making a cavity in the solvent, as well as the attractive and repulsive parts of the van der Waals interactions between the solute and the solvent. All these three non-polar solvation terms are free energies and in particular the cavitation energy should have important entropic components, representing the reorganization of the solvent around the solute. In summary, in MM/GBSA, the free energy of a state is estimated from the following sum: G = E_bnd_ + E_el_ + E_wdW_ + G_pol_ + G_np_ - TS. A 12–6 Lennard-Jones term was used to model de MM contribution. For GB, the solute dielectric constant was set to four while that of the solvent was set to 80, and the dielectric boundary was calculated using a solvent probe radius of 1.4 Å. The polar contribution is calculated using GB, and the non-polar energy is estimated by solvent accessible surface area (SASA).

### Expression of Transporter cDNA in *Xenopus laevis* Oocytes

Wild-type GlyT1 and GlyT1-M475E mutant cDNAs cloned in pSP64T vector were linearized with XbaI. Wild-type GlyT2 and GlyT2-E650M mutant cDNAs cloned in pAMV vector were linearized with ClaI. Capped GlyT1 and GlyT2 cRNAs were transcribed using SP6 and T7 polymerase, respectively (mMessage mMachine kit, Ambion Inc., TX, United States), as described (Gimenez et al., 2012). The oocytes were harvested from *Xenopus laevis* females (Xenopus Express, France) anesthetized in 0.125% (w/v) ethyl 3-aminobenzoate methanesulfonate (Sigma-Aldrich, St. Louis, MO, United States) solution in tap water. All the procedures were in accordance with the Spanish and European guidelines for the prevention of cruelty to animals. Oocytes were defolliculated by 1 h treatment with 300 U/ml collagenase (Type 1, Sigma-Aldrich, St. Louis, MO, United States) in a solution containing (in mM): 82.5 NaCl, 2 KCl, 1 MgCl_2_, 5 Hepes, pH 7.4. Fifty nanograms mRNAs were injected into stage V–VI defolliculated oocytes, which were maintained in Barth’s medium (in mM): 88 NaCl, 1 KCl, 0.33 Ca(NO_3_)_2_, 0.41 CaCl_2_, 0.82 MgSO_4_, 2.4 NaHCO_3_, and 10 HEPES, pH 7.4. Experiments were performed 3–6 days after injection.

### Two-Microelectrode Voltage-Clamp Recordings From *Xenopus laevis* Oocytes

Two electrode voltage clamp was used to measure and control the membrane potential and to monitor the capacitative currents using Axoclamp 900A, digitized using a Digidata 1440. Both instruments were controlled by pCLAMP software (Axon Instruments, Foster City, CA, United States), and the results were analyzed using Clampfit 10.3 software (Molecular Devices). Recordings from oocytes were performed at 18°C using standard micropipettes filled with 3 M KCl (resistance < 1 MΩ) (Gimenez et al., 2012). All electrophysiological recordings were performed in oocytes held at -60 mV (unless otherwise stated in the figures) using a recording solution, containing (in mM): 100 NaCl, 2 KCl, 1 CaCl_2_, 1 MgCl_2_, 10 HEPES, pH 7.4. In ion substitution experiments, Na^+^ was isosmotically replaced by choline chloride. In Cl^-^ free recordings, Cl^-^ was fully replaced using (in mM): 100 chloride gluconate, 2 KNO_3_, 1 Ca(NO_3_)_2_, 1 MgSO_4_, 10 HEPES, pH 7.4. For E_Rev_ measurements, the oocytes were inject prior to recording with 18 nl of solutions containing either glycine (1 M) alone ore glycine (1 M) and NaCl (1 M). After injection oocytes were superfused with solutions containing different external sodium concentrations (chloride and glycine external concentrations remained constant). For any given external Na^+^ concentration current-voltage (I–V) measurements were recorded at 100 μM external glycine in the absence and presence of extracellular ALX-1393 (5 μM) to yield the net glycine-evoked currents.

### Ethics Statement

This study was carried out in accordance with the principles of the Basel Declaration and recommendations of the European Community Council Directives. All biosafety procedures and animal care protocols were approved by the bioethics committee for the research from the Universidad Autónoma de Madrid (UAM), Madrid, Spain and the CBMSO Animal Experimentation Ethics Committee, Madrid, Spain.

### Data Analysis

Non-linear regression fits of experimental data and statistical analysis were performed with GraphPad Prism 6.01 (GraphPad Software Inc., San Diego, CA, United States). Bars represent S.E.M. of at least triplicate determinations. Representative experiments are shown that were repeated at least three times with equivalent results.

## Results

### GlyT2 *d*DAT Homology Model

**FIGURE 1:**
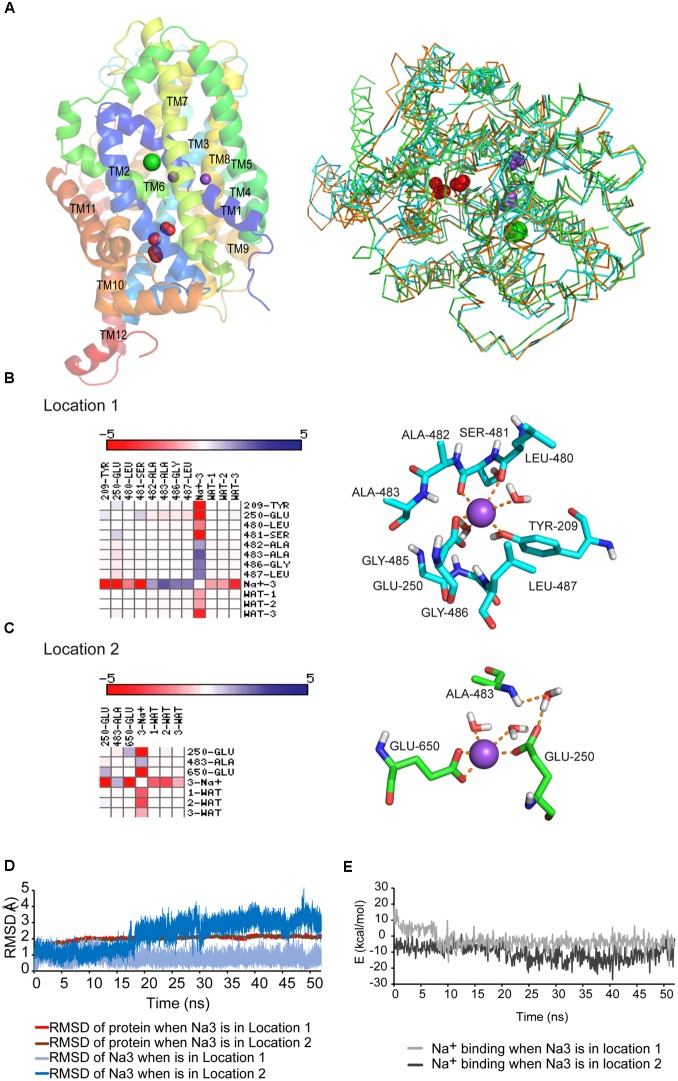
**(A)** GlyT2 dDAT homology model. Left: PyMol-generated GlyT2 lateral view is shown in a cartoon representation with conserved Na1 and Na2 as purpled spheres and chloride ion as green sphere. The water molecules found in several NSS crystals (red spheres) are surrounding the unwound portion of TM6. Right: structure alignment in PyMol of GlyT2 model, LeuT_Aa_ and dDAT crystals. 90% of the LeuT_Aa_ and dDAT crystals in PDB show 2 crystallographic water molecules in locations 1 and/or 2. The three structures are shown as ribbon, GlyT2 model is colored in orange, LeuT_Aa_ in green, and *d*DAT in cyan. The waters are depicted as red spheres and residues Glu250 and Glu650, which bind waters, are shown in stick representation. **(B)** Interaction matrix and lateral view of Na3 placed in location 1. **(C)** Interaction matrix and lateral view of Na3 placed in location 2. Cations are shown as purple spheres. Residues are shown in stick representation. **(D,E)** Root-mean-square-deviations (RMSD) **(D)** and energy profile **(E)** along the 50 ns MD simulations with sodium placed in location 1 or 2.

An outward-facing tridimensional model of GlyT2 was built using the crystalized *d*DAT structure [PDB code 4M48 (Penmatsa et al., 2013)] as template (**Figure 1A**). This DAT structure was crystalized with two bound sodium ions and one chloride ion (Penmatsa et al., 2013). In the resulting GlyT2 model the glycine binding site (S1 site) was conserved as compared to previous GlyT2 models based on LeuT_Aa_, supporting the participation of Gly212, Leu213, Tyr289, Ser481, and Thr580 in substrate recognition or translocation (Perez-Siles et al., 2011). The involvement of these residues has been validated by mutagenesis and functional assays (Rees et al., 2006; Perez-Siles et al., 2011, 2012; Carland et al., 2017). The carboxyl moiety of the substrate (glycine) coordinates the sodium ion bound at the Na1 site (Yamashita et al., 2005; Penmatsa et al., 2013). For this reason, the mutagenesis of many Na1 site coordinating residues (Ala210, Asn215, Phe478, Ser479, Leu480, Ser481, and Asn511) strongly increased Km_gly_ of glycine transport (Perez-Siles et al., 2011, 2012). Na1 and Na2 sites were conserved as well, being Na2 site contributed by Gly208, Val211, Leu576, and Asp579 (Perez-Siles et al., 2011, 2012). The residue Asp471 previously identified as a transitory cation binding site in the extracellular vestibule of GlyT2 (Perez-Siles et al., 2012) conserved the same position in the revised model. In addition, the chloride site, clearly visualized in dDAT, was conserved in the new GlyT2 model (**Supplementary Figure S1A** and see below).

The obtained GlyT2 *d*DAT homology model was inserted in a pre-equilibrated lipid bilayer box and was further optimized and tested for stability by means of 50 ns MD simulations in the presence of all the substrates: glycine, three sodium ions, and a chloride ion. In order to accommodate the third sodium ion (Na3 site of GlyT2), absent in *d*DAT, we considered two possible locations displaying crystalographic water molecules in the SLC6 crystals (Yamashita et al., 2005; Penmatsa et al., 2013; Coleman et al., 2016). There were one or two water molecules flanking the unwound TM6 portion in the crystalized structures from the family, suggesting there is enough free space in the region to hold a sodium ion (**Figure 1A** and **Supplementary Figure S1B**). In one of such locations (location 1, **Figure 1B**) sodium would be coordinated by Tyr209, Glu250, Leu480, and Ser481. The second position (location 2, **Figure 1C**), very close to the recently proposed Na3 site (Subramanian et al., 2016), would involve the side chains of Glu250 and Glu650 and the back-bone of Ala483 (**Supplementary Tables S1**, **S2**). In both cases, the complexes (protein and substrates) were stable during the 50 ns MD trajectories showing root-mean-square-deviation (RMSD) lower than 3Å in location 1, and up to 4Å in location 2 (**Figure 1D**). The RMSD changes depicted in **Figure 1D** after approximately 15 ns implied a displacement of the third Na^+^ located at position 2. The stability of the ion after relocation suggests stronger coordination. The mean calculated interaction energy value for glycine in the last ns of the dynamics was ΔE = -33.2 and -32.5 kcal/mol for structures containing Na3 in locations 1 and 2, respectively (**Supplementary Figure S2B**). The energy values for the binding of sodium in Na3 site were in kcal/mol -3.4 (location 1) and -8.85 (location 2, **Figure 1E**). This suggests a more favorable position for sodium in location 2 conceivably stabilized by the electrostatic interaction with Glu250 and Glu650 (**Figure 1C**). The stability of the glycine binding site was maintained during the MD simulations with similar RMSD values and comparable energy profiles regardless of the location occupied by the third sodium (**Supplementary Figure S2A**). Closer inspection of the glycine binding site across the simulations revealed that the use of the *d*DAT template independently of the Na3 position resulted in the emergence of new coordinating residues (**Supplementary Figures S2C,D**). The presence of Na3 in location 2 enabled the reorientation of Ala210, Val211, Gly214, Phe478, and Trp484 to the substrate (**Supplementary Tables S3**, **S4**). These amino acids were predicted (and some confirmed) (Perez-Siles et al., 2011; Carland et al., 2017) to belong to the glycine site (Rees et al., 2006; Carta et al., 2012). Besides, Tyr209 is involved in the stabilization of the unwound TM6 region and the Na2 site through a conservative backbone hydrogen bond with Ser481 within the crystals. Placing Na3 in location 1 may disturb Tyr209 interactions. Therefore, our data point to location 2 as more suitable for Na3 accommodation.

### Functional Analysis of Glu250 and Glu650 Mutants

**FIGURE 2:**
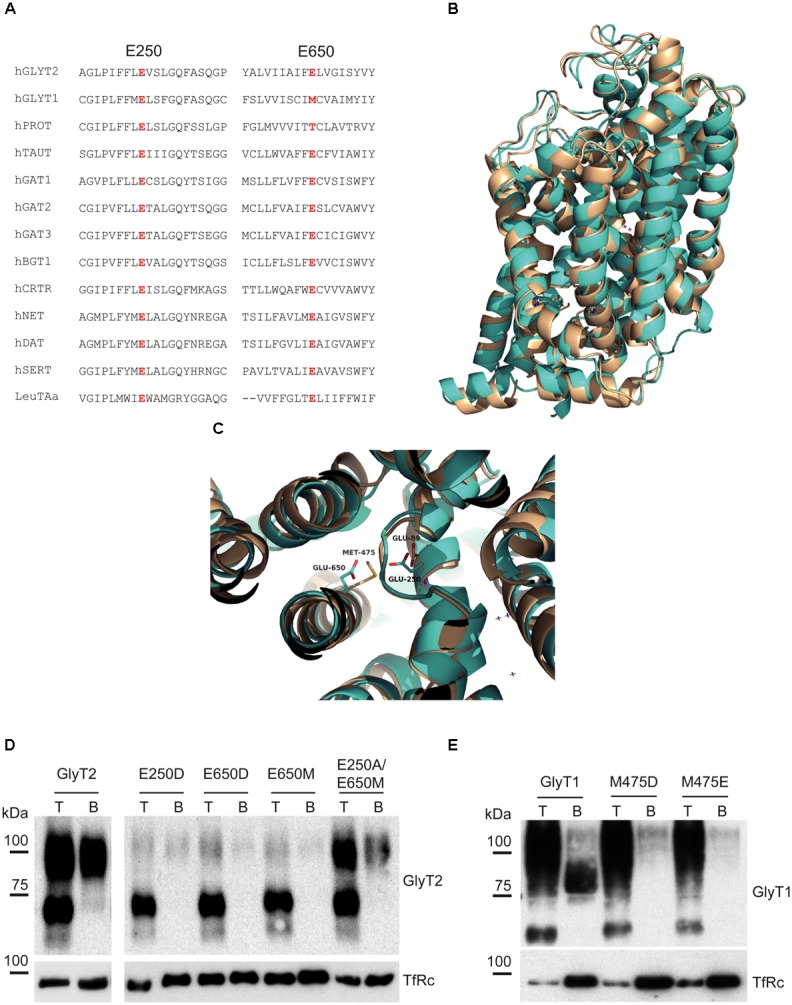
GlyT1 and GlyT2 alignment and mutant expression. **(A)** Alignment of the amino acid sequences of TM2 and TM10 of rat NSS transporters using the ClustalW program. **(B,C)** Structural alignment of modeled GlyT1 and GlyT2 showing GlyT2 Glu250 and Glu650 and GlyT1 Glu89 and Met475 in stick representation. **(D,E)** Surface expression of GlyT2 **(D)** and GlyT1 **(E)** mutants assessed by surface biotinylation. T, total transporter; B, biotinylated transporter; TfRc, transferrin receptor as loading control.

A MSA of several NSS transporters (**Figure 2A**) illustrates GlyT2 Glu250 is totally conserved within the family (including GlyT1 Glu89) but GlyT2 Glu650, although present in other members, is non-conservatively substituted by methionine (Met475) in GlyT1. The structural alignment of GlyT1 and GlyT2 models based on *d*DAT shows the voluminous Met475 reducing the free space in GlyT1 as compared to Glu650 in GlyT2 (**Figures 2B,C**). This condition, together with the absence of the coordinating oxygen in the methionine side chain might prevent the formation of a cation binding site in GlyT1. To test this hypothesis, we mutated the predicted main coordinating residues in location 2 (Glu250 and Glu650) and studied mutant properties once expressed in COS7 cells. The mutants used in this study were GlyT2-E650M and its reverse mutant GlyT1-M475E; the aspartate substitutions GlyT2-E650D and GlyT1-M475D; the GlyT2-E250D and the double mutant GlyT2-E250A/E650M. All the mutants were delivered to the plasma membrane, as assessed by surface biotinylation (**Figures 2D,E**). The main effect of the substitution in every wild-type transporter was a reduction in the plasma membrane expression and the corresponding decrease in glycine transport. One exception was the double mutant, for which the transporter fraction that reached the surface approached the level of wild-type GlyT2. Moreover, the double mutant showed no apparent phenotype (**Table 1**). Nonetheless, all the mutations were tolerated, and the levels of transport were sufficient for subsequent studies. Every mutant used in this study was properly inhibited by the corresponding specific inhibitor of GlyT1 (NFPS) or GlyT2 (ALX1393), indicating correct folding and general structure (not shown). In contrast, GlyT2 point substitutions involving Glu250 other than E250D (substitutions to A, T, and Q) were not functional, and the reciprocal mutant GlyT1-E89D was inactive despite it reached the surface. Lastly, with the exception of GlyT2-E250A/E650M, GlyT2 double mutations affecting Glu250 plus Glu650 could not be analyzed because the mutants did not reach the plasma membrane (substitutions to D/M; T/D; D/D; or T/M were assayed, not shown).

**Table 1:** Kinetic parameters of glycine transport by GlyT2 and GlyT1 mutants in transfected COS7 (150 mM NaCl).

Mutant	Vmax (nmol/mg/10 min)	*K*m (μM)	Vmax/*K*m
wtGlyT2	32.5 ± 8.7	133.5 ± 25.7	0.24 ± 0.04
E250D	14.2 ± 3.9	92.6 ± 6.7	0.16 ± 0.04
E650D	11.8 ± 4.3	66.7 ± 25.9	0.18 ± 0.03
E650M	17.7 ± 7.2	102.4 ± 33.7	0.15 ± 0.03
E250A/E650M	35.8 ± 19.1	177.2 ± 103.4	0.21 ± 0.01
wtGlyT1	114.6 ± 11.1	199.9 ± 35.0	0.62 ± 0.17
M475D	91.3 ± 0.5	630.0 ± 163.7	0.17 ± 0.04
M475E	45.6 ± 17.5	542.6 ± 168.9	0.12 ± 0.05

Kinetic parameters of glycine transport by active mutants are shown in **Table 1**. The main feature of GlyT2 single mutants was a reduction in Vmax (about 30–40% of wild-type), whereas GlyT1 mutants mainly showed increases in Km (about two to threefold). Diminished Vmax was also detected in GlyT1-M475E. The ratio Vmax/Km was reduced in every mutant: 15–40% (GlyT2 mutants) and 70–80% (GlyT1 mutants), suggesting that glycine transport was less altered by the mutations in GlyT2 than in GlyT1 background.

**FIGURE 3:**
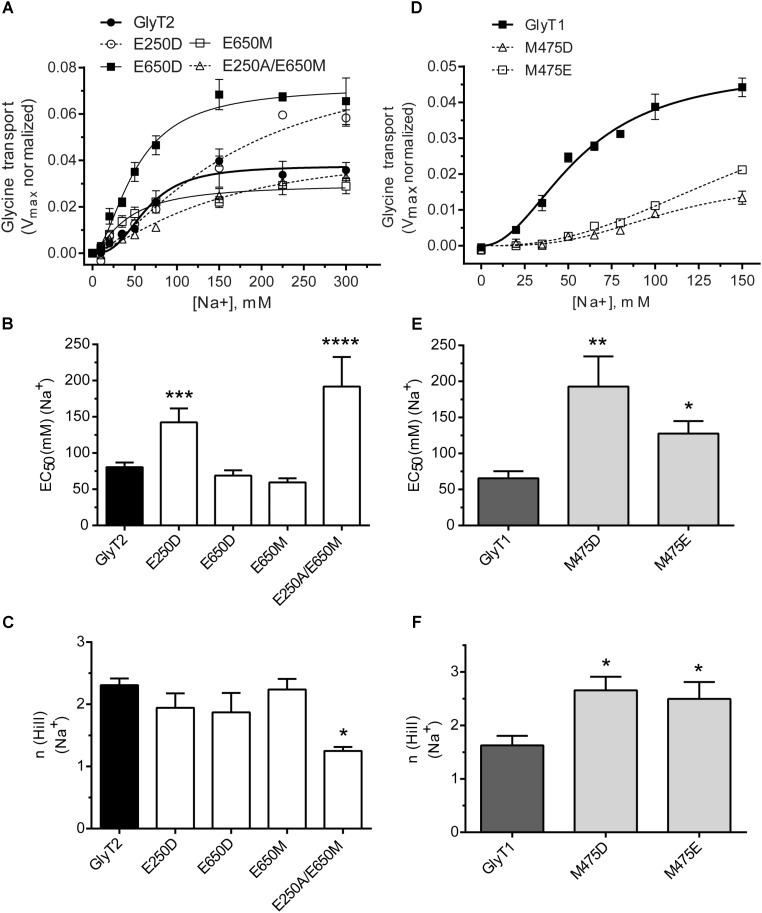
Sodium dependence of glycine transport by GlyT2 and GlyT1 substitution mutants. COS7 cells expressing the indicated transporters were assayed for glycine transport in the presence of increasing extracellular NaCl concentrations (isotonic substitution by choline chloride). Control glycine transport by wild type GlyT2 and GlyT1 were 2.0 ± 0.06 and 5.49 ± 0.26 nmol glycine/mg protein/10 min, respectively. **(A,D)** Representative experiments are shown that were repeated at least three times performed in triplicate. Glycine transport has been *V*_max_ normalized. Experimental data were fitted to the Hill equation. **(B–F)** EC_50_ for sodium and Hill coefficient were determined and analyzed by one-way ANOVA with Dunnett’s *post hoc* test, comparing with wild-type GlyT1 and GlyT2. *p* > 0.05; ^∗^*p* < 0.05; ^∗∗^*p* < 0.01; ^∗∗∗^*p* < 0.001; ^∗∗∗∗^*p* < 0.0001. For mutants nonsaturated at 150 mM NaCl, determinations up to 300 mM NaCl were performed.

The above mutants were tested for sodium dependence of glycine transport (**Figure 3**). Single or double GlyT2 mutants including Glu250 substitutions showed significantly reduced sodium affinity as well as diminished Hill coefficient that reached significance in the GlyT2-E250A/E650M double mutant (**Figures 3A–C**). For GlyT1, Met475 substitution mutants showed reduced apparent Na^+^ affinity, which was significantly lower than wild-type (EC_50Na+_ in mM: 192.8 ± 41.8, *n* = 4; 127.6 ± 17.3, *n* = 3 vs. 65.5 ± 9.8, *n* = 7 for GlyT1-M475D, GlyT1-M475E, and wild-type GlyT1). In addition, both Met475 substitution mutants showed significant increases in the Hill coefficient (*n*) (1.63 ± 0.18 vs. 2.50 ± 0.31 and 2.66 ± 0.25 for wild-type GlyT1, GlyT1-M475E, and GlyT1-M475D, respectively, **Figures 3D–F**).

### Electrophysiological Characterization of Glu-250 and Glu-650 Mutants in *Xenopus laevis* Oocytes

**FIGURE 4:**
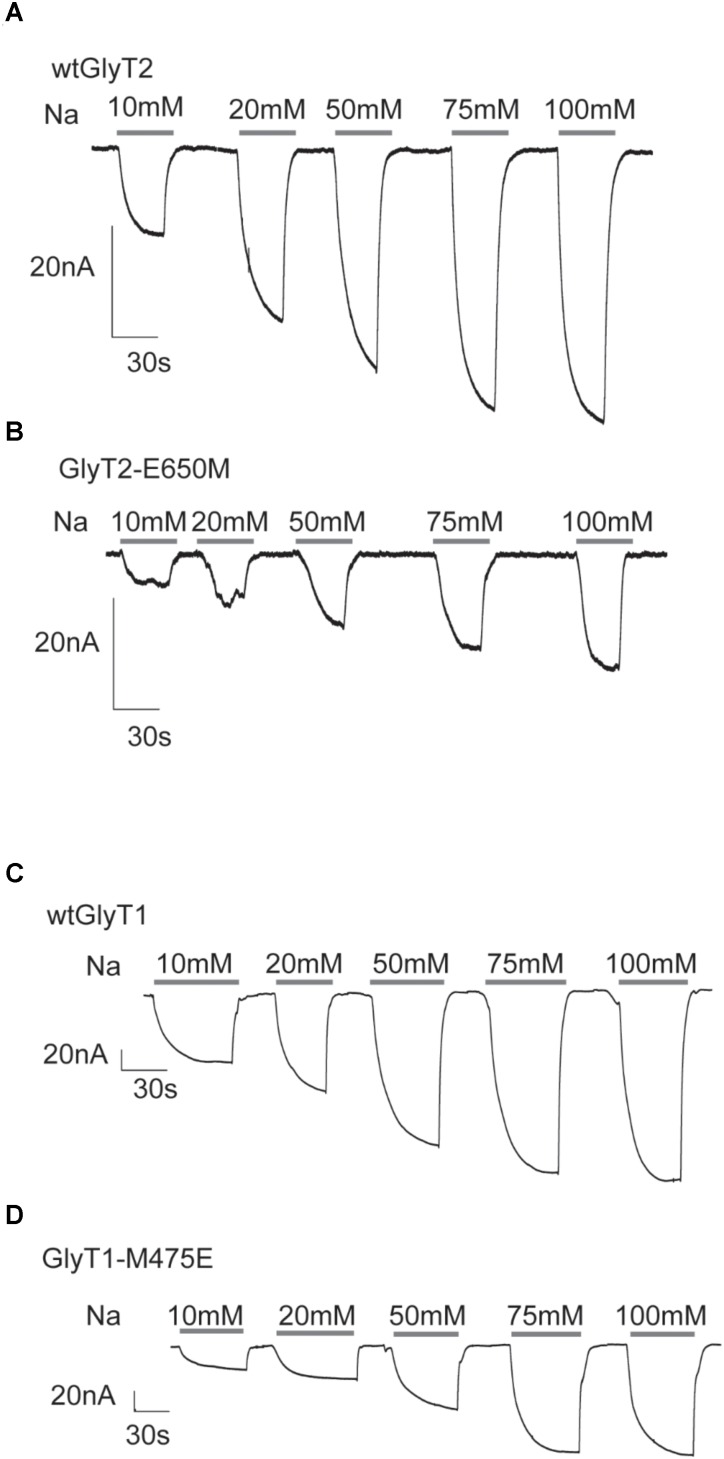
Glycine-associated currents of GlyT2 and GlyT1 substitution mutants at different external sodium concentrations. **(A–D)** Inward currents recorded at a holding potential of –60 mV elicited by application of 1 mM glycine at the indicated sodium concentrations (extracellular NaCl concentrations isotonically substituted by choline chloride) in four representative oocytes expressing the indicated transporters.

**FIGURE 5:**
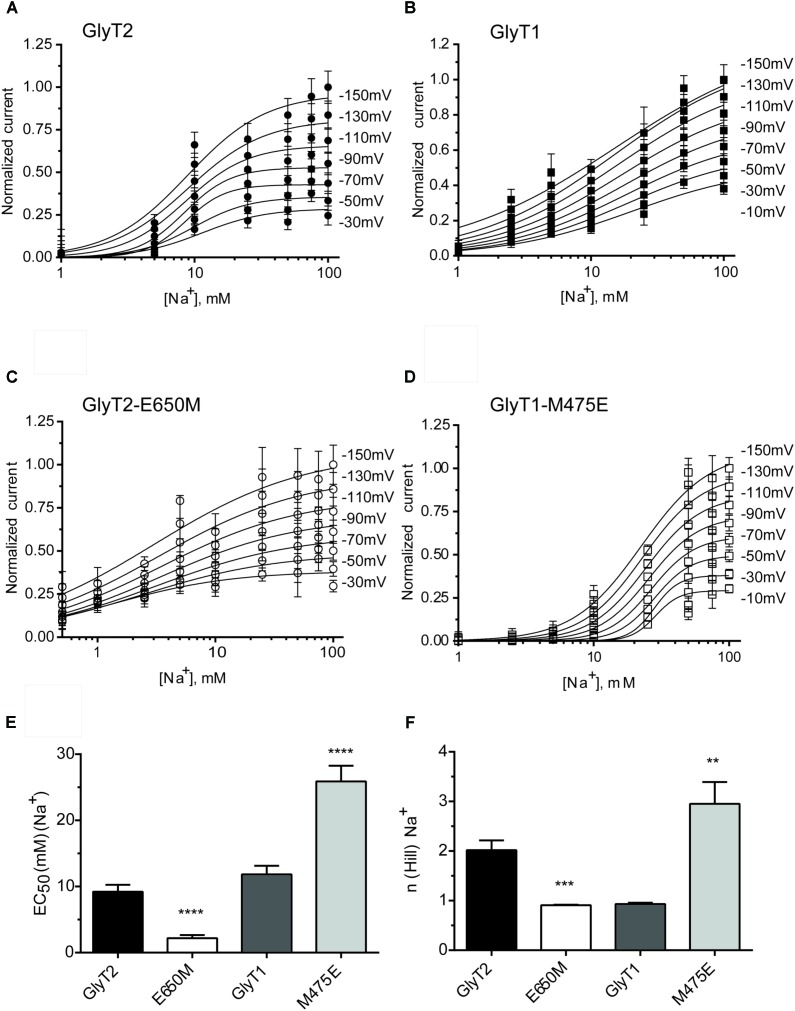
Sodium dependence of glycine-evoked steady-state currents in the oocytes expressing GlyT2 and GlyT1 wild-type and mutants. **(A–D)** At least six oocytes expressing the indicated transporters were voltage-clamped at the specified clamping potentials over the range of –150 and –10 mV and glycine-induced currents at increasing external sodium concentrations (choline substitution) were measured to estimate the EC_50_ **(E)** and Hill coefficient **(F)**. At each potential glycine-induced currents were averaged and normalized to those at –150 mV. Data were analyzed by paired *t*-test, comparing with wild-type GlyT1 or wild-type GlyT2. ^∗∗^*p* < 0.01; ^∗∗∗^*p* < 0.001; ^∗∗∗∗^*p* < 0.0001.

**FIGURE 6:**
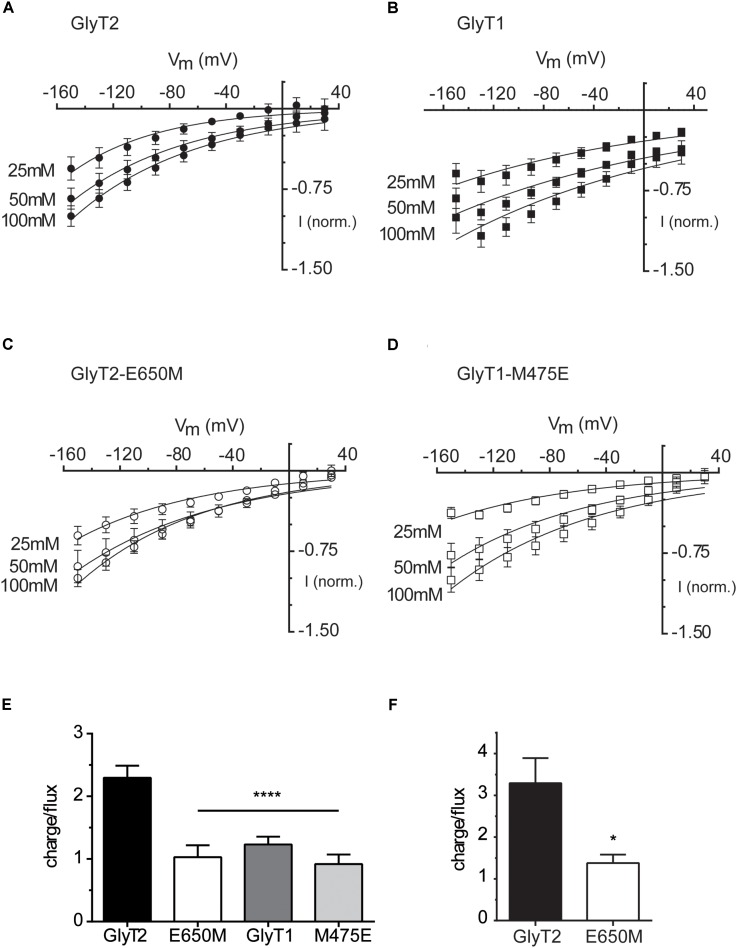
Glycine-induced steady-state currents and charge-to-flux ratios by wild-type GlyTs and mutant transporters. **(A–D)** Glycine-induced currents: the membrane voltage of oocytes expressing the indicated transporters was stepped from a holding potential of –60 mV to voltages between –150 to 30 mV in –20 mV increments. Currents in 100 mM NaCl recording solution were subtracted from those in the same medium supplemented with 1 mM Gly. At each potential glycine-induced currents were averaged and normalized to those at –150 mV. These currents were then plotted against the corresponding potential (millivolt). The data are the mean ± SEM (error bars) of at least four different oocytes. Wherever error bars are not visible, the error was smaller than the size of the symbols. The mean currents at –150 mV induced by 1 mM glycine were –67.6 ± 6.7 nA in GlyT2, –34.8 ± 2.7 nA in GlyT2-E650M, –132.1 ± 19.2 nA in GlyT1, and –77.6 ± 23.9 in GlyT1-M475E. The rectification degree has been estimated as the ratio of the slopes of the linear regression fit of the three current values at the most negative and most positive potentials in the I/V curves. The calculated rectification degree at 100 mM NaCl are 4 (GlyT2), 1.75 (GlyT2-E650M), 1.3 (GlyT1), and 1 (GlyT1-M475E). **(E,F)** charge-to-flux ratios: oocytes expressing the indicated transporters were voltage-clamped at –60 mV **(E)** or –100 mV **(F)** in 100 mM NaCl recording solution and the current induced by 30 μM radioactive glycine (0.4 Ci/mmol) was measured for 1 min. The charge moved during this time was obtained by integrating the current over time (Clampfit v. 10.3). The ratio was determined by calculating the moles of charge and dividing by the moles of radiolabeled substrate taken up as determined from scintillation counting after correction by the values for non-injected oocytes. The data are given in mean ± SEM (error bars) of at least four different oocytes. ^∗∗∗∗^*p* < 0.0001 one-way ANOVA with Bonferroni’s multiple comparison test, ^∗^*p* < 0.05 Mann–Whitney test.

We explored the electrophysiological properties of the above mutants assayed in *Xenopus* oocytes. Some of the mutants that were active in COS7 cells did not generate currents (GlyT2-E250D, GlyT2-E650D, GlyT1-M475D, and GlyT2-E250A/E650M, not shown). In contrast, the application of 1 mM glycine to oocytes expressing GlyT2-E650M, GlyT1-M475E, or the wild-type transporters clamped at -60 mV induced the appearance of robust inward currents of increased amplitude as the external sodium concentration was elevated and reached saturation (**Figures 4A–D**). Glycine-evoked currents were measured at different holding potentials over the range of –150 and –10 mV (**Figure 5**). The Na^+^ dependence of the normalized currents fit to the Hill equation for individual potentials allowed estimating EC_50_ and Hill coefficient. The estimated parameters in oocytes at saturating glycine concentrations (1 mM) replicated the tendency in COS7 cells although the reduction in EC_50_ and Hill coefficient of the E650M mutant reached significance, perhaps due to the saturating glycine concentrations that can be used in oocytes but not in cells where basal glycine transport due to low affinity GlyTs obligates using low μM concentrations. On the contrary, the GlyT1-M475E mutant kept displaying higher EC_50_ and Hill values than GlyT1 (**Figures 5E,F**). The glycine-induced steady-state currents by oocytes expressing wild-type transporters were non-reversing inward currents (**Figures 6A,B**), reflecting electrogenic Na^+^-coupled glycine influx. As reported, GlyT2 currents showed higher inward rectification than those of GlyT1 (Lopez-Corcuera et al., 1998; Roux and Supplisson, 2000). Quantification of the rectification degree indicated the GlyT2-E650M mutant displayed reduced rectification as compared to GlyT2 wild-type (**Figures 6A,C**), although non-significant difference with GlyT1was exhibited by the GlyT1-M475E mutant (**Figures 6B,D**). No reversion of the currents was observed for any transporter, suggesting they are transport-associated stoichiometric currents. We compared the time integral of the glycine-induced current (charge) with the simultaneous [^3^H]-glycine uptake (flux) for each transporter to estimate the net number of charges translocated when the oocytes were clamped at hyperpolarizing potentials (**Figure 6E**). As previously reported, GlyT2 moves two positive charges per transported glycine molecule (Roux and Supplisson, 2000; Vandenberg et al., 2007) and, accordingly, it gave a charge-to-flux ratio of 2.30 ± 0.19, *n* = 10 at -60 mV. At this potential, our measurements on GlyT2-E650M gave a ratio of 1.03 ± 0.19, *n* = 6, what was significantly lower to wild-type GlyT2 and similar to that of wild-type GlyT1 (1.23 ± 0.13, *n* = 17), which translocates 1 charge per transport cycle (Roux and Supplisson, 2000; Vandenberg et al., 2007; Perez-Siles et al., 2012; López-Corcuera et al., 2017) (**Figure 6E**). Interestingly, the charge-to-flux ratio of GlyT2-E650M was also significantly lower than wild-type GlyT2 in oocytes clamped at -100 mV (3.29 ± 0.6, *n* = 4 for GlyT2 and 1.38 ± 0.2, *n* = 5 for E650M) (**Figure 6F**), in agreement with a fixed stoichiometry of glycine transport (Sonders et al., 1997). These data suggest that the substitution of Glu650 by methionine impairs the ability of GlyT2 to hold the third sodium ion. Conversely, the acidic substitution of Met475 in GlyT1 does not increase sodium coupling since the charge-to-flux ratio for GlyT1-M475E mutant (0.91 ± 0.15, *n* = 8) indicates the mutant is able to translocate only one charge per transport cycle as wild-type GlyT1. Although the estimated Hill coefficient and the charge-to-flux ratio suggested GlyT2-E650M may cotransport a lower number of Na^+^ ions, a further approach to the direct evaluation of its stoichiometry was obtained by determining the equilibrium potential of the transporter (E_T_) or E_Rev_ as a function of the external sodium concentration ([Na ^+^]o). The GlyT2 E_Rev_ is the membrane potential at which transport is in thermodynamic equilibrium (not inward or outward transport) and it depends on the ionic and substrate electrochemical gradients as indicated by the thermodynamic relationship:

$${\text{E}}_{\text{T}}=\frac{2.3\text{R T}}{({\text{n}}_{\text{Na}}-{\text{n}}_{\text{CI}})\text{F}}\log\left(\frac{{\left[{\text{Na}}^{+}\right]}_{\text{o}}^{{\text{n}}_{\text{NA}}}{\left[{\text{CI}}^{-}\right]}_{\text{o}}^{{\text{n}}_{\text{CI}}}{\left[\text{Gly}\right]}_{\text{o}}}{{\left[{\text{Na}}^{+}\right]}_{\text{i}}^{{\text{n}}_{\text{Na}}}{\left[{\text{CI}}^{-}\right]}_{\text{i}}^{{\text{n}}_{\text{CI}}}{\left[\text{Gly}\right]}_{\text{i}}}\right)$$

**FIGURE 7:**
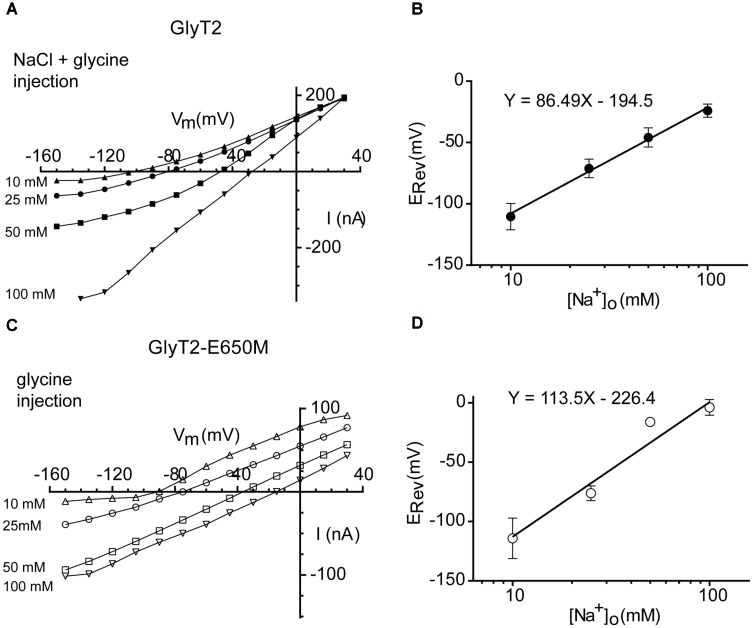
Reversal potential of wild-type and GlyT2-E650M mutant-mediated currents as a function of the external Na^+^ concentration. **(A,B)** GlyT2 oocytes injected with 18 nM of glycine and NaCl: current-voltage relationships **(A)** and E_Rev_ plotted as a function of the log [Na^+^]o **(B)**. **(C,D)** GlyT2-E650M oocytes injected with 18 nM of glycine: current-voltage relationships **(C)** and E_Rev_ plotted as a function of the log [Na^+^]o **(D)**. Linear regression equation is presented in the graphs.

To obtain measurements of the E_Rev_ of the wild-type and mutant transporters, both inward and outward currents must be measured in the same oocyte, so it is necessary to decrease the driving force. It has been demonstrated that coinjection of glycine and NaCl is necessary to evoke outward currents in GlyT2-expressing oocytes whereas injection of glycine alone was sufficient to evoke outward currents in GlyT1 (Roux and Supplisson, 2000). When we set up the experimental conditions, we confirmed the injection of glycine and NaCl was necessary for GlyT2 reversion but glycine alone was sufficient to evoke outward currents in GlyT2-E650M, so that we established these conditions for the determination of the E_Rev_ of the two transporters. As the extracellular Na^+^ concentration was increased, E_Rev_ shifted to more positive membrane potentials (**Figures 7A,C**). A plot of E_Rev_ as a function of log [Na^+^]o revealed a slope of 86.49 ± 11.05 mV (*n* = 7–9) for a tenfold change in [Na^+^]o for GlyT2 (**Figure 7B**). This value almost coincides with the predicted change in E_Rev_ at 18°C (86.50 mV for *z*_T_= 2), and gives *n*_Na_ = 2.99 for GlyT2. For GlyT2-E650M, the slope was 113.50 ± 12.10 mV (*n* = 5–13), very close to the theoretical prediction of 115.36 mV for 2Na^+^:1Cl^-^:1gly stoichiometry, giving *n*_Na_= 1.97 for *z*_T_ = 1 (**Figure 7D**). These data suggest that GlyT2-E650M mutant could translocate one less Na^+^ ion during the transport cycle.

### Molecular Dynamics Simulations of GlyT2-E650M

To analyze the effect of E650M mutation on the modeled GlyT2 structure, we performed 50 ns MD simulation of the mutated structure (**Supplementary Figure S3**). The dynamics was started with Na3 placed in location 2. During the first ns, Na3 jumped from its initial position and moved toward Glu250, losing the interaction with the position 650 where the glutamate was mutated to methionine (**Supplementary Figures S3A,C**). The mutant dynamics started with unfavorable positive initial energy values indicating repulsive energy, which become less repulsive as the dynamics progressed due to the entry of water molecules solvating Na^+^ in location 2 (**Supplementary Figure S3B**). However, energy values were always very close to zero. After approximately 20 ns of simulations, repulsive interactions appeared between Na^+^ and the surrounding residues. There was an important lose in electrostatic interaction energy (ΔE = -0.6 vs. -8.85 kcal/mol, see above), indicating the absence of Glu650 makes the binding of Na3 in location two non-favorable. Although the structural and energetic stability of the binding of substrate and the rest of ions to the mutant transporter were comparable to that of the wild-type GlyT2 model, according to the ability of the mutant to display about 50% wild-type glycine transport, it is evident the mutation energetically influences the putative Na3 site.

### Allosteric Effects of Putative Na3 Site Mutants

**FIGURE 8:**
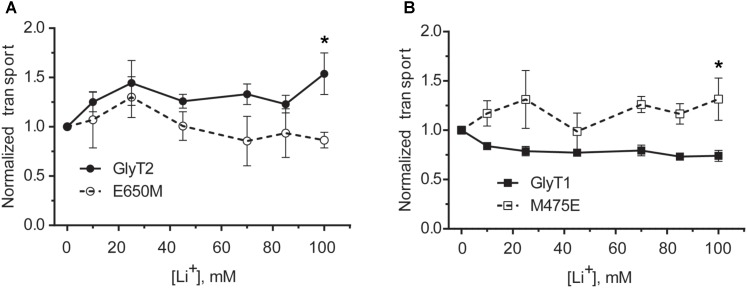
Lithium dependence of glycine transport of wild-type GlyTs and mutant transporters. **(A,B)** COS7 cells expressing wild-type GlyT2 or GlyT2-E650M **(A)** or wild-type GlyT1 or GlyT1-M475E **(B)** were assayed for ^3^[H]-glycine transport in HBS containing 30 mM NaCl concentration in every point and the indicated increased LiCl concentration up to 150 mM that was reached by isotonic supplementation with choline chloride. ^∗^*p* < 0.05 Mann–Whitney test.

The above data suggest Glu650 (together with Glu250) is a key residue for the sodium coordination in the putative GlyT2 Na3 site. These two glutamates align with Glu419 (TM10) and Glu62 (TM2) in LeuT_Aa_, which are residues sensitive to the differential ion-specific conformational changes transmitted through the TM10-TM6 interface upon the binding of sodium or lithium (Zhao et al., 2011). Since lithium exerts differential effects in the activity of the GlyTs (Perez-Siles et al., 2011, 2012), we analyzed the response to lithium ion on glycine transport by the putative Na3 site mutants. In agreement with our previous data, **Figures 8A,B** shows that the effect of lithium in COS7 cells expressing GlyT2 was stimulatory, whereas it exerted a noncompetitive inhibitory effect on GlyT1 transport. Conversely, the GlyT2-E650M and the GlyT1-M475E mutants displayed an inverted tendency as compared to their wild-types. These data suggest that the ion-specific local interactions in the vicinity of these particular TM10 residues can be emulated by the substitutions. It is also possible that the conformational changes induced at TM10 can propagate to the ion-binding sites (Kantcheva et al., 2013).

### Chloride Dependence of Putative Na3 Site Mutants

**FIGURE 9:**
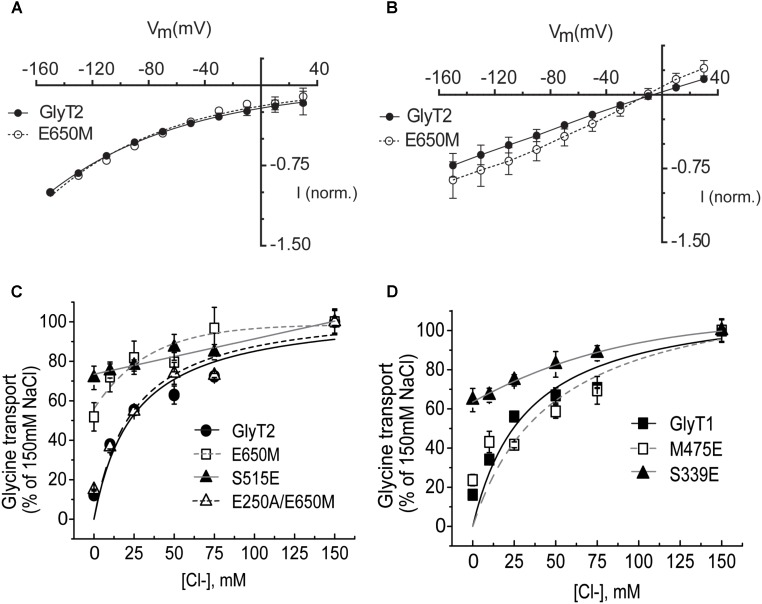
Chloride dependence of glycine-induced steady-state currents and glycine transport by wild-type GlyTs and mutant transporters. **(A,B)** Glycine-induced currents: the membrane voltage of oocytes expressing the indicated transporters was stepped from a holding potential of –60 mV to voltages between –150 to 30 mV in –20 mV increments. Currents in 100 mM NaCl recording solution **(A)** or in chloride-free solution (described in material and methods) **(B)** were subtracted from those in the same medium supplemented with 1 mM Gly. At each potential glycine-induced currents were averaged and normalized to those at –150 mV. **(C,D)** COS7 cells expressing the indicated transporters were assayed for glycine transport in the presence of increasing extracellular NaCl concentrations (isotonic substitution by sodium gluconate). Control glycine transport by wild-type GlyT2 and GlyT1 was 3.3 ± 0.3 and 2.6 ± 0.3 nmol glycine/mg protein/10 min, respectively. Representative experiments are shown repeated at least in three separated experiments performed in quadruplicate. Experimental data were fitted to the Michaelis–Menten equation.

The role of the TM6-TM10 interface in the propagation of the conformational changes induced by Na^+^ binding (Zhao et al., 2011) prompted us to investigate the effect of mutations in the putative Na3 site on the chloride dependence. We measured the current-voltage relation of the wild-type GlyT2 and the GlyT2-E650M mutant in regular recording medium containing 100 mM NaCl (**Figure 9A**) or the same medium where the chloride was isosmotically substituted (nominally zero chloride, see Materials and Methods) (**Figure 9B**). Normalized wild-type and mutant currents were almost identical when measured in external medium containing NaCl (**Figure 9A**). In the nominal absence of chloride, outward currents were evident in the two transporters though the amplitude was higher in the mutant (**Figure 9B**). The E_Rev_ around -10 mV suggests proton or cation diffusion in the absence of chloride (Mager et al., 1994; Sonders et al., 1997; Longpré et al., 2010). To further confirm this observation, we measured glycine transport by COS7 cells expressing GlyT2-E650M mutant in the presence of increasing chloride concentrations (NaCl isosmotically substituted by sodium gluconate). As depicted in **Figure 9C**, transport by the mutant showed about 50% reduction at zero-chloride, and slightly increased at higher Cl^-^ concentrations. For comparison we analyzed GlyT2-S515E. In this mutant, the serine that aligns with Glu290 in LeuT, which is the residue responsible for the chloride independence of transport by prokaryote NSS members, is substituted to glutamate (Zomot et al., 2007). The introduction of a negative charged amino acid at or near the two sodium-binding sites of several NSS transporters renders transport chloride-independent (Zomot et al., 2007; Ben-Yona et al., 2011; Kantcheva et al., 2013). In fact, GlyT2-S515E and the equivalent mutation in GlyT1 (S339E) almost abolished the chloride dependence of transport (**Figures 9C,D**). Glycine transport by GlyT2-E650M was mostly unaltered in the presence of increasing chloride concentrations similarly to GlyT2-S515E and opposite to wild-type GlyT2. However, this feature was not observed for the reverse mutant GlyT1-M475E whose hyperbolic dependence on chloride was very similar from that of wild-type and differed from that of GlyT1-S339E (Km_Cl-_ 33 ± 6 and 49 ± 4 mM for GlyT1 and GlyT1-M475E, respectively, **Figure 9D**). These results suggest the E650M mutation in GlyT2, but not the reverse mutation M475E in GlyT1, generates allosteric effects on the chloride-binding site. We hypothesized the removal of Glu650 could leave unpaired the Glu250, which may fulfill pKa requirements to affect the chloride site. If this hypothesis was correct, the simultaneous removal of Glu650 and Glu250 could restore chloride dependence of transport. We, therefore, analyzed the chloride dependence of glycine transport by the double mutant GlyT2-E250A/E650M and found it was indistinguishable from that of wild-type (Km_Cl-_ 25 ± 4 and 24 ± 3 mM for GlyT2 and GlyT2-E250A/E650M, respectively), indicating the simultaneous mutation of the two glutamates completely rescued the chloride dependence of transport to the wild-type phenotype (**Figure 9C**).

## Discussion

The recent resolution of eukaryotic crystals of the SLC6 family has provided new three-dimensional structures for modeling the GlyTs. We previously validated GlyT models using the prokaryotic LeuT_Aa_ template (PDB 2A65) (Yamashita et al., 2005) using site directed mutagenesis and confirmed the conservation of the substrate and two sodium sites (Na1 and Na2) in the GlyTs (Perez-Siles et al., 2011, 2012). By profiting the availability of crystalized structures with higher homology to the GlyTs, here we have generated and validated homology models using the *Drosophila melanogaster* DAT crystal as a template (PDB 4M48) (Penmatsa et al., 2013). A *d*DAT-based modeled structure was lately used by others to propose the location of the GlyT2 Na3 site, absent in the template, by MD simulations in which the Na^+^ ion was placed at the homologous position to that of the Na1′ site of the betaine transporter BetP. Several queries were raised on the stability of this proposed sodium binding site since the eventually obtained sodium site was spatially separated from the BetP Na1′ site due to ion jumping along the simulation (Subramanian et al., 2016).

In order to settle the location of the third Na^+^ site in GlyT2, we initially tested two possible Na3 accommodations in the *d*DAT-based GlyT2 model. These positions displayed crystallographic water molecules in the available NSS crystals (Yamashita et al., 2005; Penmatsa et al., 2013; Coleman et al., 2016). The MD simulations we performed indicated higher stability and lower interaction energy values for Na3 binding when located close to the proposed Na3 site (Subramanian et al., 2016), although our simulations excluded Met278 (IL1) and Trp265 (TM3), which were included in that study perhaps due to some incipient transition toward the inward conformation during simulations. Among the residues contributing to the putative Na3 site, the unique non-conservative substitution between GlyT2 (3 Na^+^ sites) and GlyT1 (2 Na^+^ sites) is Glu650 (TM10) in GlyT2, which is replaced by a methionine (Met475) in GlyT1. Among the NSS transporters most are coupled to two Na^+^ ions although there are some coupled to one Na^+^ ion. In SERT, it has been proven that Na^+^ binds to Na1 and Na2 and generates conformational changes in both of them, but only the Na^+^ ion bound to Na2 is translocated (Felts et al., 2014). This indicates that the presence of a sodium site may not have consequences in stoichiometry, thus eliminating the requirement of unique residues forming the third Na^+^ site. In GlyT2, the residues surrounding the site could generate an environment that facilitates Na^+^ binding. As this environment shows much higher homology with GlyT1 than with other SLC6 members, GlyT1 could be specifically prevented from Na^+^ binding by substituting the sodium-binding oxygen in the position 650/475 side chain by a methionine.

By site-directed mutagenesis and examination, the sodium dependence of glycine transport by the mutants, together with the electrophysiological analysis of mutant currents in *Xenopus laevis* oocytes, we obtained evidences that suggest the Na3 ion is held by Glu650 and Glu250 in GlyT2. The charge-to-flux ratio for the GlyT2-E650M mutant was reduced as compared to wild-type GlyT2 to a value comparable to that of GlyT1 even at strongly hyperpolarizing potentials, thus, suggesting the GlyT2 mutant had reduced sodium coupling compatible with a non-functional Na3 site. Since other alterations could result in a change in the charge-to-flux ratio, we determined the E_Rev_ as a function of the external sodium concentration, which agrees with the prediction for n_Na_ = 2 in GlyT2-E650M mutant in contrast to wild-type GlyT2. For these measurements glycine and NaCl were injected into the oocytes. GlyT2 has a kinetic constraint for reverse transport assuring an asymmetry in glycine fluxes which is essential to maintain high levels of neurotransmitter inside the presynaptic neurons. Internal Na^+^ binding may be critical in GlyT2 since the injection of glycine alone failed to activate outward currents. For that reason, it has been proposed that the third intracellular Na^+^ site has lower affinity and limits reverse transport (Roux and Supplisson, 2000). In our experimental conditions, the reverse transport in GlyT2-E650M mutant was detected by injecting glycine alone thus increasing intracellular glycine levels. Since the proposed third sodium site is more internal than Na1 and Na2 sites, the loss of this site could facilitate reversal transport. The fact that GlyT1 has a methionine instead of Glu650 could be related with the absence of such a limitation for reverse transport. In good agreement, the rectification of the I/V curves that might reflect the tendency of GlyT2 to operate in the forward direction and therefore to show an asymmetry of transport function, is much less evident in the GlyT2-E650M mutant. However, the rectification degree obtained for the GlyT1-M475E mutant was not significantly different from that of GlyT1, according with the incapability of this mutant to bind a Na3 ion. Although the E_Rev_ as a function of external glycine and chloride must be determined to confirm the reduction in translocated sodium ions, the data suggest Glu650 is a key residue in the maintenance of the transport coupling.

In sodium dependence assays, we estimated the Hill coefficient (*n*), which serves as a reliable indicator of transport stoichiometry only if a very high degree of cooperativity exists between the binding sites. When all of the cotransported Na^+^ ions bind with a positive cooperativity (*n* > 1) and there are not transporters partially filled with Na^+^, the rate of transport varies with extracellular Na^+^ concentration depending on *n*_Na_. In that cases fitting to Hill equation will provide a value for *n* close to n_Na_ (Prinz, 2010). We found some discrepancies between the value of *n* derived from kinetic analysis for GlyT2-E650M and GlyT1-M475E and our charge-to flux determinations. One possibility is that, the transport assays in COS7 cells under non-saturating glycine concentrations did not achieve the maximum transport rate to obtain a reliable *n* fitting to Hill equation. That is solved in oocyte assays where glycine is at saturating concentrations. However, the increase in GlyT1-M475E Hill coefficient is also evident in oocytes pointing to the possibility that multiple ions must bind to the transporter for translocation to occur, but only one of the ions is actually translocated. In this case, kinetics might indicate *n* > 2 even though only two Na^+^ ions were cotransported (Rudnick, 1998; Willford et al., 2015).

The behavior of the GlyTs seems to differ from that of other members of the SLC6 family such as the monoamine transporters, which show uncoupled or leak currents either dependent or independent of substrate (Mager et al., 1994; Sonders et al., 1997; Felts et al., 2014). Moreover, the deletion of TM10 residues in the GABA transporter GAT1 leads to the appearance of GABA-induced outward currents and increased charge-to-flux ratios indicating uncoupled transport (Dayan et al., 2017). Conversely, all the TM10 mutants analyzed in this study showed non-reversing inward currents compatible with coupled glycine influx, and the charge-to-flux ratio of GlyT2-E650M mutant was lower than wild-type at strongly negative membrane potentials (-100 mV), though some increase in the values for both transporters was detected (**Figure 6**). Furthermore, in the absence of substrate, we did not measure significant currents in the presence of sodium or lithium both in the wild types and the mutants. Moreover, [^3^H]glycine transport was always sodium-driven and not lithium-driven. However, in the absence of chloride in the external medium, we could measure leak currents both for wild-type GlyT2 and to a higher extent for the Glyt2-E650M mutant. This suggests there is an uncoupled current blocked by chloride in the wild-type that is increased in the mutant. Although no specifically tested, the E_Rev_ of this current close to -10 mV suggests the involvement of protons or other cations (Mager et al., 1994; Sonders et al., 1997; Longpré et al., 2010). This feature is remarkable since it has been reported that chloride independence of NSS transporters is associated to proton antiport (Zhao et al., 2010). The relation of this current with stoichiometric transport in the presence of chloride is presently unknown.

Although predicted in other members of the NSS transporters (Zhao et al., 2010; Kantcheva et al., 2013; Zhao and Noskov, 2013; Dayan et al., 2017), our study unveils the robust allosteric properties of the TM10 region containing Glu650 in GlyT2 but also Met475 in GlyT1. The substitution of these TM10 residues affects sodium cooperativity, especially increased in the GlyT1-M475E mutant. Besides, the wild-type transport responses to lithium ion are inverted by the substitutions. This suggests the local interactions generated at the Na2 site, the unique sodium site where lithium binding may sustain transport in the GlyTs, are altered (Perez-Siles et al., 2011). The inversion of the lithium responses in the mutants suggests that only when glutamate is present at this TM10 position the conformational changes induced by lithium binding to the Na2 site are favorable to transport, as takes place in wild-type GlyT2 (Perez-Siles et al., 2012). Our results confer this TM10 region of the GlyTs a role in the allosteric propagation of the cation binding energy for the translocation of substrates. Indeed, the allosteric connection of this region with the S1 (and putative S2) site has been validated in other NSS members (Zhao et al., 2011). The strong cooperativity among the sodium sites suggests this region has ideal properties for the location of the Na3 site in GlyT2 although it might also sustain a role as a modulatory site or a Na^+^ exit site.

The substitution of Glu650 in GlyT2 by a neutral amino acid (Met) present in GlyT1 makes the glycine transport by the mutant almost chloride-independent. This condition is reversed by the simultaneous substitution of Glu650 and Glu250 by neutral amino acids in the double mutant GlyT2-E650M-E250A. Since it has been demonstrated that the introduction of a negatively charged amino acid, near the ion-binding sites of the NSS transporters renders the transport chloride-independent (Zomot et al., 2007; Ben-Yona et al., 2011; Kantcheva et al., 2013), we propose it is the negative charge of the unpaired Glu250 what alters the chloride dependence in the GlyT2-E650M mutant. We hypothesize that in the wild-type GlyT2 Glu250 and Glu650 interact with Na^+^ and in its absence with water molecules as in other NSS such as LeuT_Aa_ (Zhao et al., 2011). This maintains the two glutamates paired by an electrostatic interaction. In the GlyT2-E650M mutant, the charge of the residue in 650 disappears and Glu250 is unpaired. This causes the introduction of a negative charge near the ion-binding sites what alters the chloride dependence of GlyT2. In the double mutant GlyT2-E650M-E250A, the charges from the two glutamates disappear and there is not remaining unpaired negative charge that may alter the chloride dependence. For this reason, the double mutant is chloride-dependent comparable to wild type. The role of Glu250 as the residue that undertakes protonation-deprotonation within the transport cycle of the GlyT2-E650M mutant is reinforced by the fact that Glu250 is included within the interaction network connecting the ion binding sites (Ben-Yona et al., 2011; Kantcheva et al., 2013). Indeed our data could not discard a proton antiport instead chloride symport by GlyT2-E650M. Conversely, GlyT1 transport remains dependent on chloride regardless the equivalent position to Glu650 contains glutamate or methionine, suggesting not only the charge but the overall transporter structure or the network connecting the ion binding sites is differential in GlyT1. Our results unveil the interaction between Glu650 and Glu250 in GlyT2 has differential properties as compared to GlyT1. This supports an additional role for this region in GlyT2 such as a putative sodium-binding site. In addition, our data suggest the chloride sites in GlyT1 and GlyT2 display unique properties as has been demonstrated for their extracellular vestibules (Nunez et al., 2000; Perez-Siles et al., 2012). The residues coordinating the chloride ion in eukaryotic NSS transporters connect with the external gate through a hydrogen bond network supporting a role for chloride in gate closing (Zomot et al., 2007; Kantcheva et al., 2013). We propose Glu250 and Glu650 have a relevant role in the control of the closure of the transporter external gate by holding a sodium ion either transiently or stoichiometrically coupled.

## Author Contributions

BL-C and CA designed the experiments. CB-M and EN performed the transport experiments and performed the electrophysiological analysis. AP, DA, and HdS performed the computational studies. CB-M, AP, and BL-C analyzed the data. AP and BL-C wrote the manuscript.

## Conflict of Interest Statement

The authors declare that the research was conducted in the absence of any commercial or financial relationships that could be construed as a potential conflict of interest.

## Footnotes

1 http://biophysics.cs.vt.edu/H++

2 http://www.charmm-gui.org/

## Abbreviations

ALX1393: *O*-[(2-Benzyloxyphenyl-3-flurophenyl)methyl]-L-serine
DAT: dopamine transporter
EL: external loop, E_Rev,_ reversal potential
GBSA: generalized born surface area
GlyT: glycine transporter
HBS: HEPES-buffered saline
IL: internal loop
LeuT_Aa_: leucine transporter from *Aequifex aeolicus*
MD: molecular dynamics
MM: molecular mechanics
MSA: multiple sequence alignment
NSS: neurotransmitter–sodium symporter
RMSD: root-mean-square deviation
SERT: serotonin transporter
SLC: solute carrier
TM: transmembrane domain
