# Supplementary figures and images for: Modification of a Putative Third Sodium Site in the Glycine Transporter GlyT2 Influences the Chloride Dependence of Substrate Transport
Source: Front Mol Neurosci. 2018 Sep 24;11:347. doi: 10.3389/fnmol.2018.00347 (PMC6166138; doi:10.3389/fnmol.2018.00347)

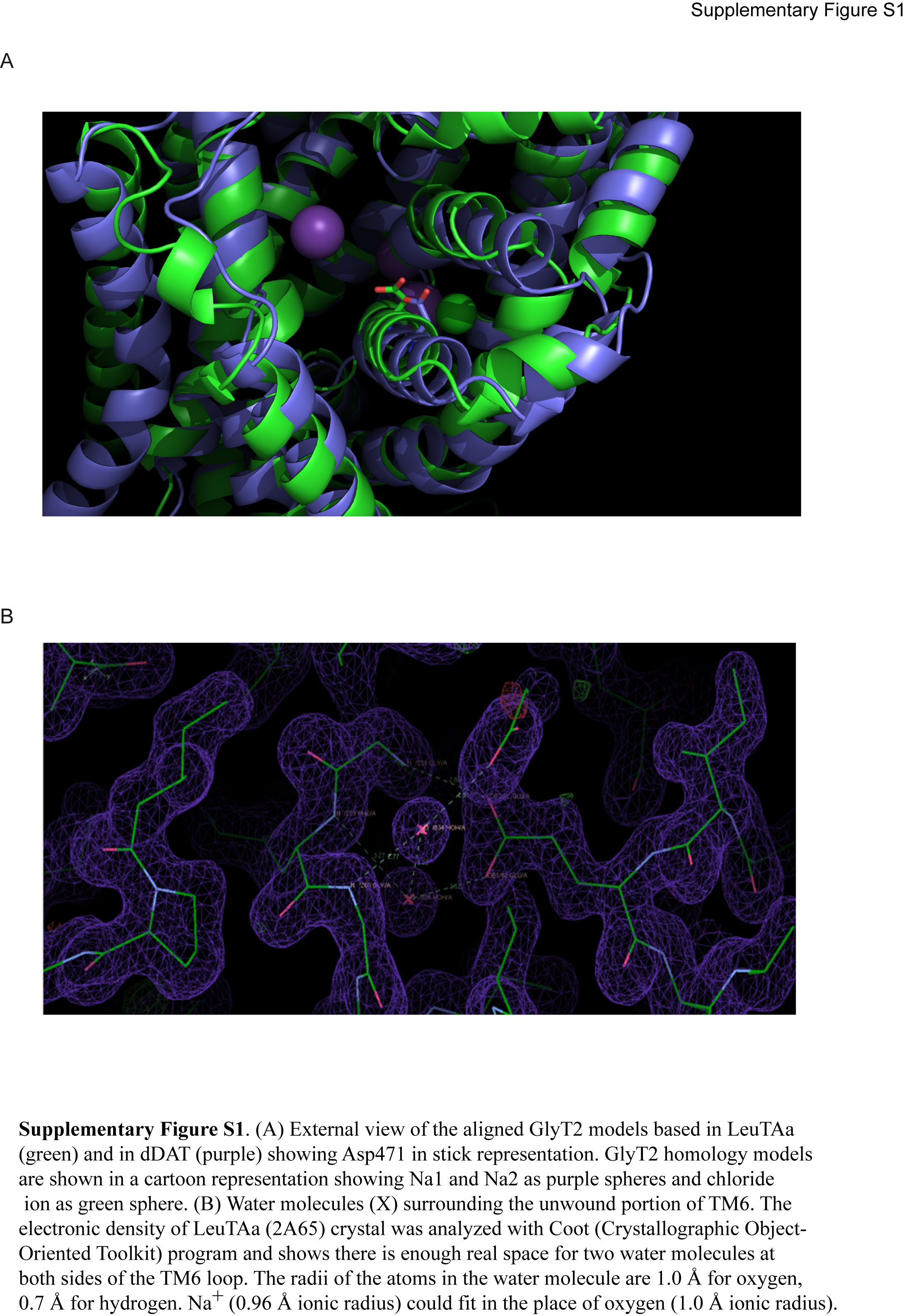

Supplement: Supplementary file 1 [file Image_1.jpg]

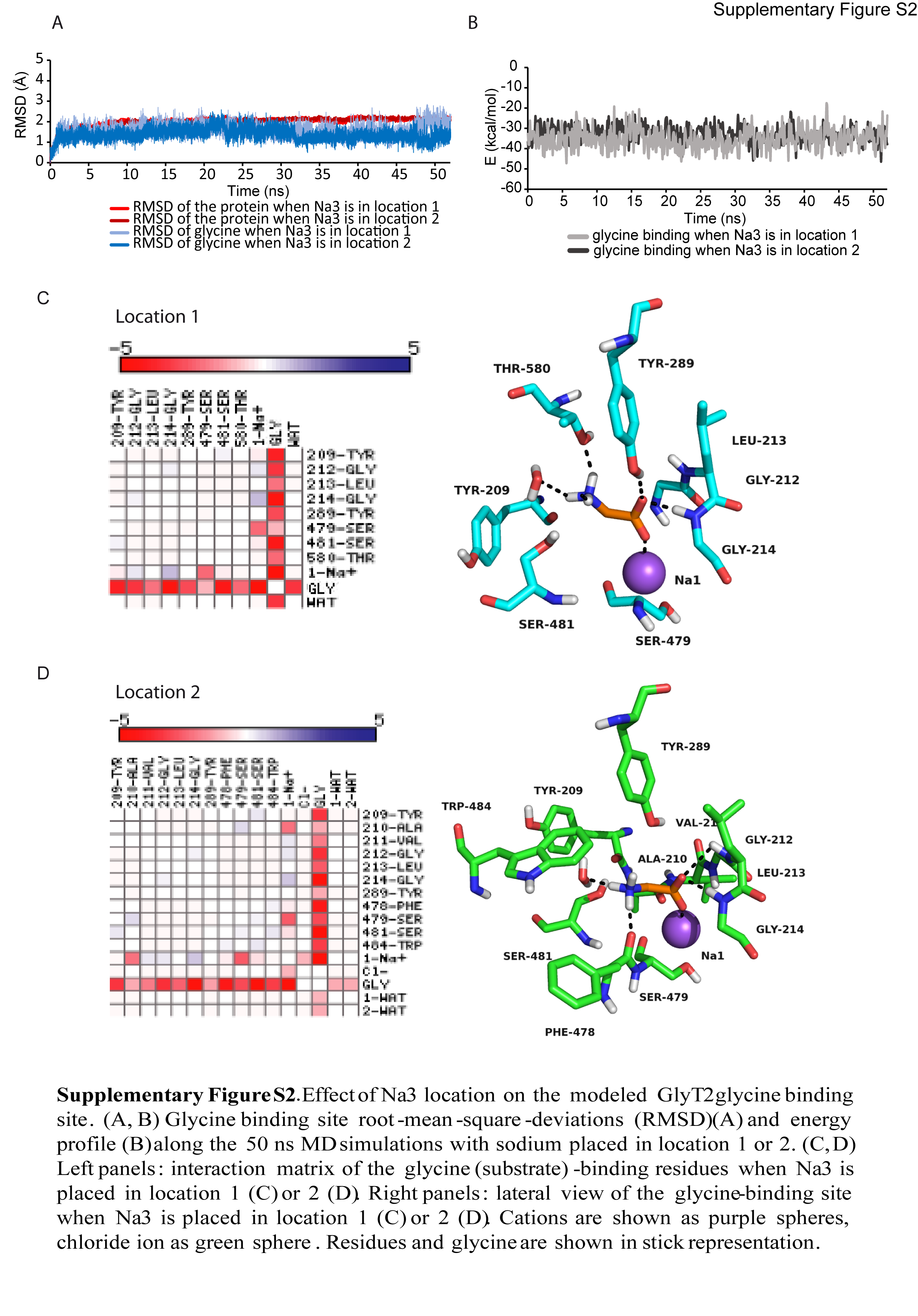

Supplement: Supplementary file 2 [file Image_2.TIF]

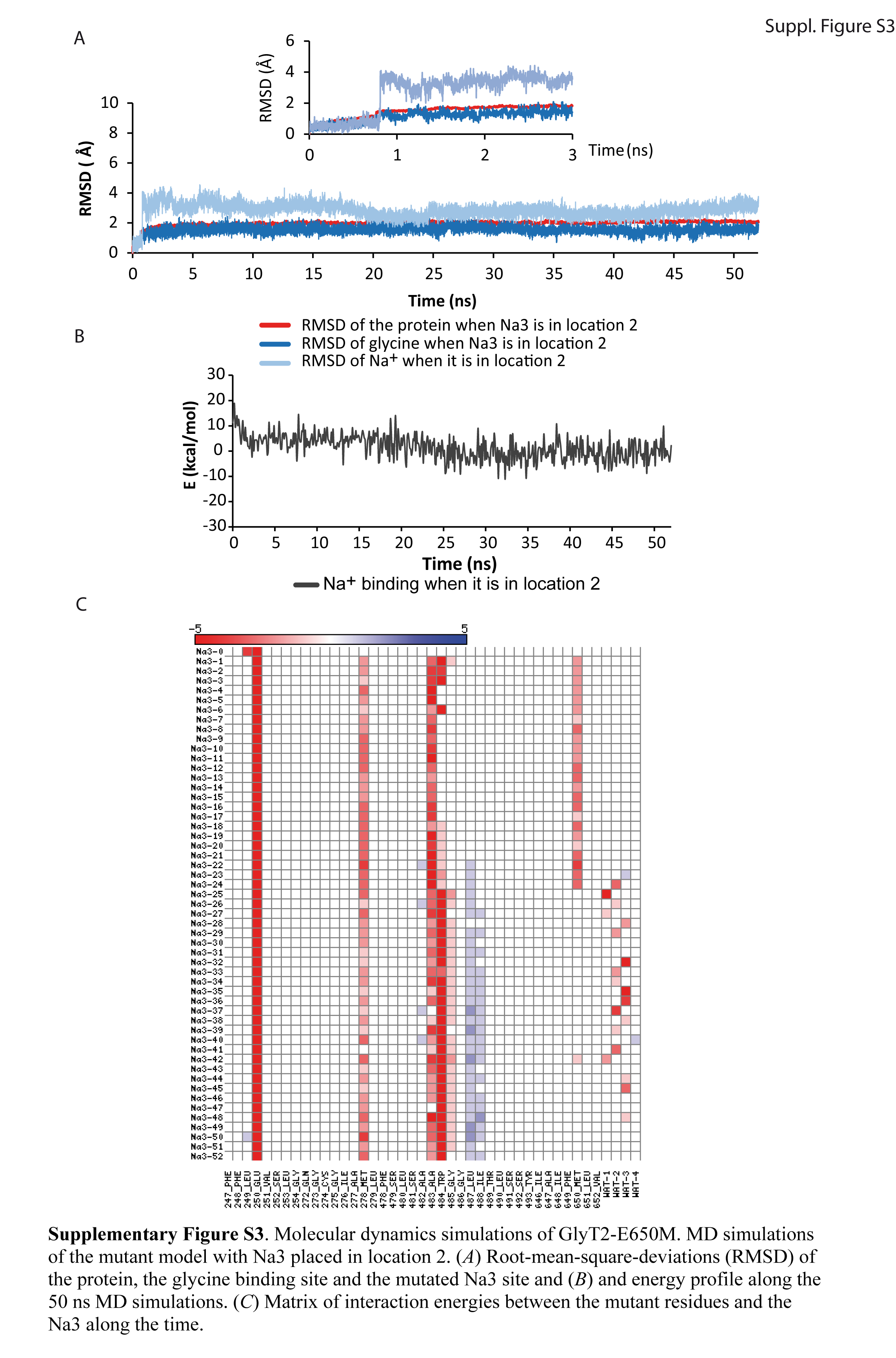

Supplement: Supplementary file 3 [file Image_3.TIF]

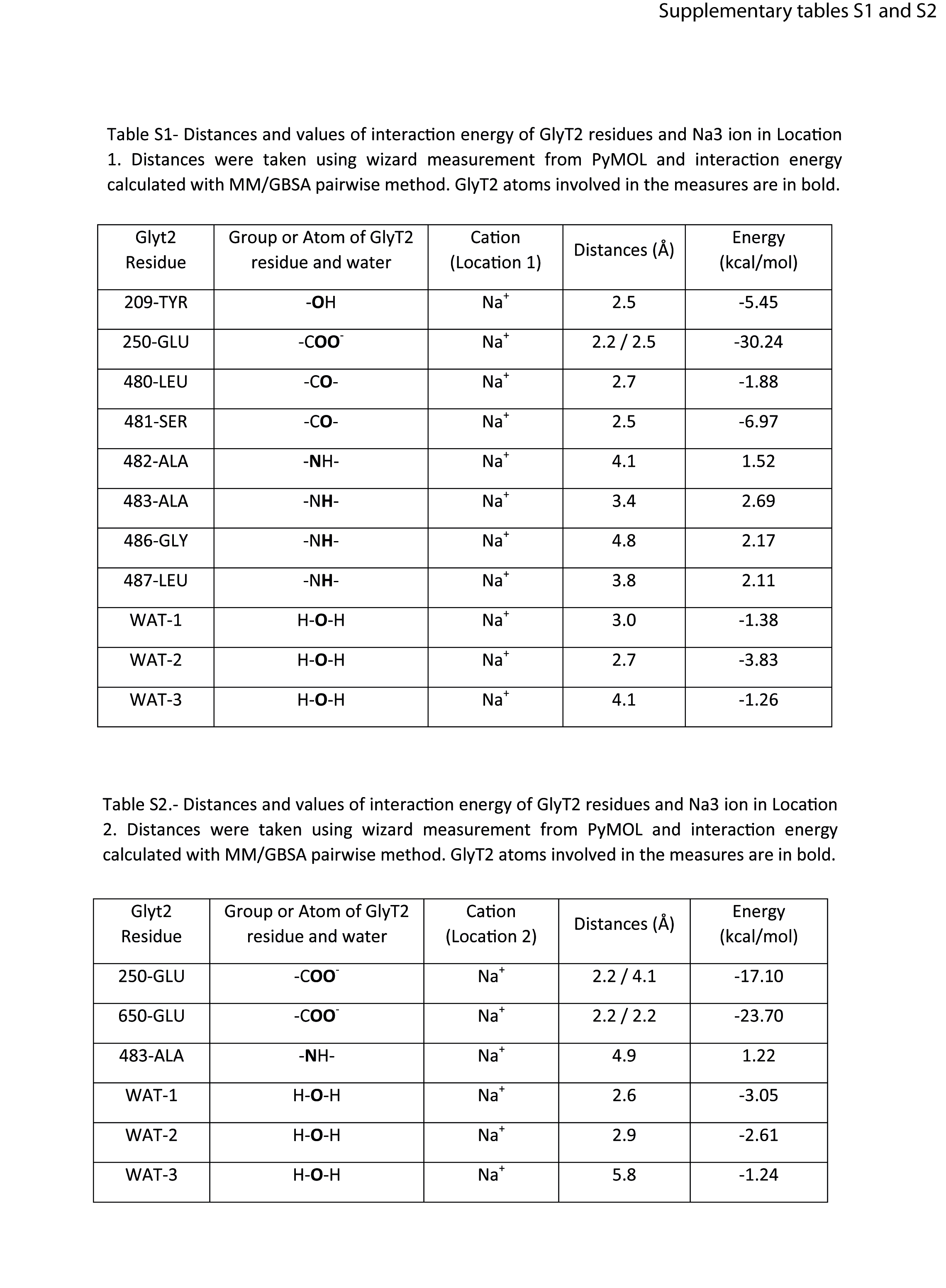

Supplement: Supplementary file 4 [file Image_4.tif]

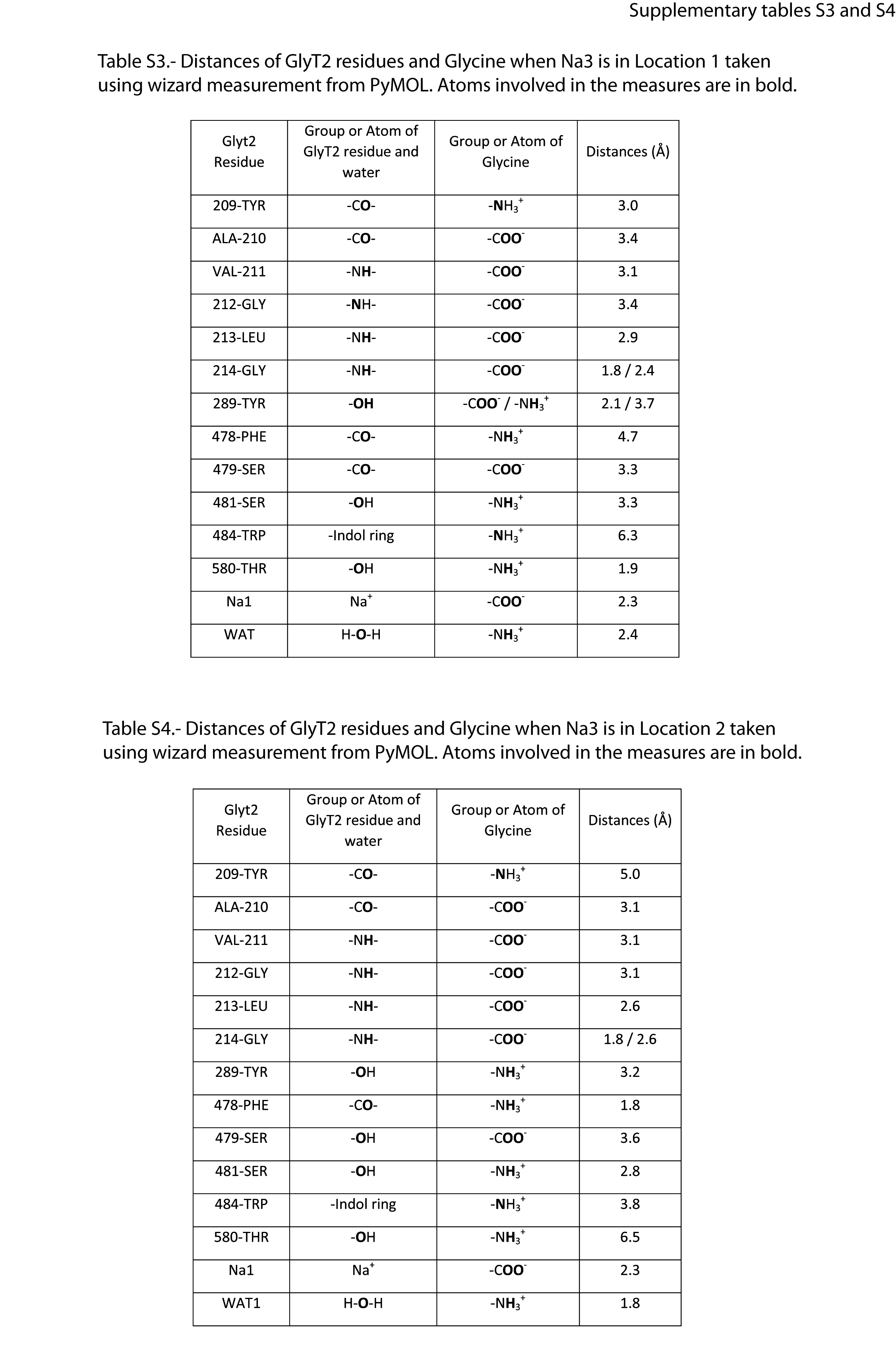

Supplement: Supplementary file 5 [file Image_5.tif]
